# Do patient and practice characteristics confound age-group differences in preferences for general practice care? A quantitative study

**DOI:** 10.1186/1471-2296-14-90

**Published:** 2013-06-25

**Authors:** Willemijn A de Graaf-Ruizendaal, Annette J Berendsen, Dolf de Boer, Dinny H de Bakker

**Affiliations:** 1 Department of primary care organization, NIVEL: Netherlands Institute for Health Service Research, PO Box 1568, 3500 BN Utrecht, The Netherlands; 2 Department of General Practice, University of Groningen, University Medical Center Groningen, 9713 AV Groningen, The Netherlands; 3 Department of quality of care, NIVEL: Netherlands Institute for Health Service Research, the Dutch Centre for Consumer Experience in Health Care housed at NIVEL, PO Box 1568, 3500 BN Utrecht, The Netherlands; 4 Department of Social and behavioural science, Tranzo Tilburg University, PO Box 90153, 5000 LE Tilburg, The Netherlands

**Keywords:** Preferences, Elderly, GP care

## Abstract

**Background:**

Previous research showed inconsistent results regarding the relationship between the age of patients and preference statements regarding GP care. This study investigates whether elderly patients have different preference scores and ranking orders concerning 58 preference statements for GP care than younger patients. Moreover, this study examines whether patient characteristics and practice location may confound the relationship between age and the categorisation of a preference score as very important.

**Methods:**

Data of the Consumer Quality Index GP Care were used, which were collected in 32 general practices in the Netherlands. The rank order and preference score were calculated for 58 preference statements for four age groups (0–30, 31–50, 51–74, 75 years and older). Using chi-square tests and logistic regression analyses, it was investigated whether a significant relationship between age and preference score was confounded by patient characteristics and practice location.

**Results:**

Elderly patients did not have a significant different ranking order for the preference statements than the other three age groups (*r* = 0.0193; *p* = 0.41). However, in 53% of the statements significant differences were found in preference score between the four age groups. Elderly patients categorized significantly less preference statements as ‘very important’. In most cases, the significant relationships were not confounded by gender, education, perceived health, the number of GP contacts and location of the GP practice.

**Conclusion:**

The preferences of elderly patients for GP care concern the same items as younger patients. However, their preferences are less strong, which cannot be ascribed to gender, education, perceived health, the number of GP contacts and practice location.

## Background

In the next 30 years, an increase in the demand for primary care is to be expected due to an ageing population [1-3]. Already, elderly patients have a substantially higher contact rate with general practice care than younger patients [4]. Primary health care must be able to adapt to the health care needs of the elderly, which are different from younger patients, to ensure the well-being of older people [1].

Moreover, primary care should address the preferences and the views of older patients [1,5], as differences in health care needs may lead to differences in preferences regarding health care [6]. Indeed, De Boer et al. found that patient groups categorised by health problem differed in their preferences for quality aspects of care [7]. Greater insight into elderly people’s preferences regarding primary care can help to make primary care more responsive to the needs of the elderly [5,6].

Substantial research has been conducted into the preferences of patients regarding quality aspects of GP care [5,6,8-13]. The influence of age on the preferences regarding GP care showed different magnitudes [6,9,10]. Moreover, one study did not find any relationship between age and preference scores. This study concluded that the results for patients’ preferences are mixed and that ‘the reason for this is unclear and may relate to a number of factors’ [11].

Even though the outcomes regarding the influence of age on preference scores differ widely, little research has been conducted into the factors which may influence these differences. In a Dutch study, Jung et al. stated that it was not age but the number of GP contact moments which had the greatest influence on preference scores. Other direct effects on preference scores were found for level of education, gender, and health status [9]. In addition to age, a systematic literature review identified a direct effect on preferences of level of education, health status, gender, family situation and utilisation of health care services [6]. Despite these direct effects on preference scores, the review did not elaborate on the possible effects on the relationship between age and preferences. To our knowledge, only two studies have elaborated on the influence of some of the above factors on the relationship between age and preference score [10,12]. However, they did so only for three preference statements regarding GP care.

In this study, we investigate whether elderly patients have different preferences concerning 58 preference statements for GP care than younger patients and examines whether gender, education, perceived health, health care use and degree of urbanisation may confound the relationship between age en preference score. These characteristics have been shown to have a major influence on the preferences for GP care [6,9]. The relationship between age and preferences regarding GP care is of special interest because older patients are more dependent on others, have a higher health care use, have a lower health status, and suffer more from chronic diseases than younger patients [3].

## Methods

### Data collection and response

Data of the Consumer Quality Index GP care (CQI GP care) were used, which were collected for the development of this instrument between 2005–2007 in 32 GP practices in the Netherlands with a total of more than 16,000 patients [14]. The practices involved were located in both rural and urban areas. Every resident in the Netherlands is registered with a GP. For all patients registered at one of these practices, name, address, date of birth and gender were extracted. Using random sampling a questionnaire was sent in name of the GP to patients from every GP practice (n = 32). One practice was situated in an disadvantage area. To compensate for the expected low response rate for this practice, 150 questionnaires were sent. One practice had a very small patient population and therefore no questionnaires were sent. The total amount of questionnaires sent was n = 3,150.

The CQI is a Dutch valid instrument to measure patient experiences and preferences regarding health care [15]. It is based on two other types of surveys: the American CAHPS (Consumer Assessment of Healthcare Providers and Systems) [16,17] and the Dutch QUOTE (QUality Of care Through the patients’ Eyes) [18-20]. The CQ-index is characterized by its disease-specific and provider-specific focus as well as the assessment of patient priorities, which both derive from the QUOTE. The lay-out, response scales and standardized sampling, data collection, analysis and presentation adopted for the QC-index were taken from CAHPS. The CQ-index has been declared to be the national standard for measuring patient experiences and performance indicators of quality of care are frequently based on the CQ-index [21,22].

The questionnaire contained questions regarding the respondents’ characteristics according to the CQI method [15] and 58 preference statements regarding GP care and the other health care providers (OHCP) in the GP practice, such as the practice nurse. The statements covered subjects such as communication, accessibility, affection, care from other health care providers such as an assistant, specialised or diabetes nurse and/or practice nurse), organisation, patient-centred care, cooperation and expertise [14]. Patients could answer on a four-point scale which ranged from ‘not important’, ‘reasonable important’, ‘important’ to ‘very important’. To address avoidance of scale extreme, especially amongst the oldest age group, the response scale are small and value labels were added to the response categories.

The questionnaire was filled in both by patients who had and by patients who had not visited a GP in the previous year. Despite the fact that those patients did not visit the GP in the previous year, they presumably have experiences with visiting the GP and therefore their preferences regarding GP care remain relevant and important.

### Statistical analysis

Patients were categorised in four age groups (0–30, 30–51, 51–75 and 75 years and older). Subsequently, a rank order was calculated for every preference statement based on the mean score of the preference statements for every age group (scores 1–4). Next, the percentage of patients who found a preference statement ‘very important’ (preference score) was calculated for every preference statement for the four age groups. Therefore, the 58 preference statements were dichotomised (0 = ‘not very important’ to’ important’, 1 = ‘very important’). The mean number of statements which were categorized as ‘very important’ was calculated for every age group. Using Students’ t-test it was calculated whether the mean preference score differed significantly between the age groups and with spearman correlation the association between the different age groups and the rank order was calculated.

To analyse whether there were significant differences in the percentage of patients who found a statement ‘very important’ (preference score) between the four age groups, a chi-square test was conducted for every statement (n = 58). To analyse which age groups differed significantly on ‘preference score’, the chi-square tests were repeated for the statements with a significant *p*-value (*p* < 0.05) for every possible combination of two age groups.

Subsequently, logistic regression was used to analyse whether there was still a significant relationship between age and preference score after gender (1 = female) and education (1 = low, 2 = average, 3 = high), perceived health (0 = less than good health and 1 = good health) and GP contact (0 = less than 5 contact moments and 1 = 5 or more contact moments), and degree of urbanisation (0 = rural, 1 = urban) had been entered. If in the fourth model there was still a significant relationship between age and preference score, we defined that the above-mentioned factors did not confound the significant relationship between age and preference score. We were interested on the effects of the factors on the relationship between age and preference score and not on their main effects. The analyses were carried out using STATA (version 10, 2009, STATACorp, College Station Texas).

## Results

### Response and demographics of the subgroups

The number of questionnaires sent amounted to 3,150. A total of 89 questionnaires were returned undeliverable. The net response was 60.7% (n = 1,858). For 35 respondents age was unknown and they were therefore excluded from the analysis (n = 1,823). The patient characteristics and the practice characteristic for the four age groups are shown in Table 1. For the four age groups, the chi-square tests showed significant differences in education (*p* < 0.001), gender (*p* < 0.001), perceived health (*p* < 0.001), number of GP contacts (*p* < 0.001) and urbanisation of the practice location (*p* < 0.001).

**Table 1:** Patient and practice characteristics for the respondents (n = 1823) in the four age groups

	**Patients 0–30 years (n = 283)**		**Patients 30–51 years (n = 633)**		**Patients 51–75 years (n = 700)**		**Patients 75 years and older (n = 207)**	
Mean age	21.4	SD = 6.3	40.8	SD = 5.9	60.8	SD = 6.5	80.74	SD = 4.4
	n	%	n	%	n	%	n	%
Education								
- unknown	14	5.0	17	2.7	47	6.7	21	10.1
- low	84	29.7	152	24.1	300	42.9	128	61.8
- medium	128	45.2	259	46.6	256	36.6	47	22.7
- high	57	20.1	169	26.7	97	13.9	11	5.3
Gender								
- unknown	2	0.7	0	0	1	0.1	0	0
- male	182	64.3	223	35.2	319	45.6	70	33.8
- female	99	34.9	410	64.8	380	54.3	137	66.2
Perceived health								
- unknown	0	0	6	0.9	13	1.9	2	1.0
- bad	2	0.7	13	2.1	22	3.1	8	3.9
- reasonable	27	9.5	94	14.8	176	25.1	85	41.1
- good	156	55.1	377	59.6	409	58.4	100	48.3
- very good	66	23.3	106	16.7	57	8.1	10	4.8
- excellent	32	11.3	37	5.8	23	3.3	2	0.1
GP contact rate								
- unknown	8	2.8	27	4.3	52	7.4	15	7.2
- 0	55	19.4	68	10.7	82	11.7	11	5.3
- 1	51	18.0	94	14.8	82	11.7	9	4.3
- 2-4	108	38.2	254	40.1	243	34.7	55	26.6
- 5-9	51	18.1	144	22.7	166	23.7	81	39.1
- 10 or more	10	3.5	146	7.3	75	10.7	36	17.4
Urbanisation practice location								
- unknown	0	0	11	1.7	23	3.3	7	3.4
- rural	152	53.7	341	53.9	301	43.0	80	38.6
- urban	131	46.3	281	44.4	376	53.7	120	58.0

### Preference scores and rank order

Table 2 shows the different preference statements (n = 58), the percentage of respondents from the four age groups who found the preference statements ‘very important’, the rank order and significant differences between the age groups in the preference scores. The preference statements were ranked according to the rank score of the patient group ‘75 years and older’. In general, the preference statements with the ten highest and lowest scores were the same for the four age groups. There was no significant difference in rank order between the four age groups. According to the Spearman rank test, the relationship between the respondents’ age and the mean score given to the preference statements was non-significant (*r* = 0.0193; *p* = 0.41). The preference statements with the three highest scores for patients ‘75 years and older’ were ‘good expertise of GP’, ‘no conflicting information from OHCP and GP’ and ‘good cooperation between OHCP and GP’.

**Table 2:** Preference scores and ranking order for the 58 preference statements for the four age groups

	**1. Patients 0–30 years**		**2. Patients 30–51 years**		**3. Patients 51–75 years**		**4. Patients 75 years and older**	
**Preference statements**	**% Very important**	**Rank order**	**% Very important**	**Rank order**	**% Very important**	**Rank order**	**% Very important**	**Rank order**
Good expertise of GP	68.9	2	74.0	1	73.6	1	67.8	1
HCP does not give conflicting information*	59.2 ^4^	5	60.3 ^4^	4	58.5 ^4^	4	48.3 ^1 2 3^	2
Good cooperation between O*HCP* and GP*	56.1 ^4^	7	56.4 ^4^	5	58.0 ^4^	3	44.9 ^1 2 3^	3
GP gives understandable explanation regarding results	55.1 ^4^	6	53.1 ^4^	6	54.1 ^4^	5	44.9 ^1 2 3^	4
GP takes me seriously**	64.0 ^3 4^	3	60.4 ^3^^4^	3	51.6 ^1^^2^^4^	6	43.2 ^1 2 3^	5
Privacy in examination room**	69.9 ^4^	1	66.7 ^4^	2	64.8 ^4^	2	47.1 ^1 2 3^	6
GP must give information on side-effects medicine**	26.2 ^2 3 4^	30	34.4 ^1^^4^	30	37.3 ^1^	20	44.2 ^1 2^	7
OHCP redirects in time**	54.5 ^4^	9	53.0 ^4^	7	54.1 ^4^	7	38.8 ^1 2 3^	8
GP listens carefully**	55.1 ^3^^4^	4	50.5 ^3 4^	8	41.1 ^1^^2^^4^	10	32.7 ^1 2 3^	9
GP must give information regarding different treatments	43.3	13	42.9	10	44.5 ^4^	8	34.6 ^3^	10
OHCP must have good expertise	40.9	18	42.2	15	43.1 ^4^	13	34.3 ^3^	11
Good diagnosis of assistant	39.9	17	45.7	12	44.2	12	39.1	12
Good accessibility of practice	46.6 ^3^	15	39.8	18	39.3 ^1^	15	38.8	13
Treatment should reduce problems	33.0	21	30.9 ^3^	29	36.8 ^2^	16	30.7	14
GP gives understandable explanation**	42.8 ^3 4^	14	40.2 ^4^	13	35.8 ^1^	14	28.8 ^1^^2^	15
GP needs to tell me what I want to know**	50.2 ^3 4^	10	43.6 ^4^	9	42.0 ^1^^4^	9	32.4 ^1 2 3^	16
Helpful staff	33.6	19	33.5	17	30.3	17	26.2	17
GP Practice must be clean**	40.1 ^2 4^	20	31.5 ^1^	24	35.6 ^4^	18	26.2 ^1 3^	18
Quick consult with own GP	21.9 ^3^	33	27.1	32	28.7 ^1^	27	26.1	19
OHCP must take me seriously**	39.2 ^3 4^	16	35.2 ^3 4^	16	29.1 ^1^^2^^4^	19	19.8 ^1 2 3^	20
GP has attention for personal situation	31.5	32	33.3 ^4^	27	30.6	31	25.0 ^2^	21
OHCP must give understandable explanation	28.6 ^4^	22	25.8	28	23.5	29	19.0 ^1^	22
Possibility to call AHGPC*	16.3 ^2 3 4^	39	22.8 ^1^^3^	38	30.4 ^1^^2^	34	27.7 ^1^	23
Consultation within 24 hours**	30.0 ^3^	24	36.2 ^3 4^	22	43.7 ^1^^2 4^	11	27.1 ^2 3^	24
Good assistance on telephone	24.4	23	25.0 ^4^	26	22.8	38	18.4 ^2^	25
Respect of GP for patient**	54.4 ^2 3 4^	8	43.1 ^1^^3^^4^	11	30.2 ^1^^2^^4^	24	22.6 ^1 2 3^	26
GP is prepared to talk regarding mistakes*	24.4 ^2 3^	29	32.0 ^1^	23	35.4 ^1^	22	30.9	27
GP must motivate advice	22.1	34	21.6	34	24.1	35	23.5	28
Good cooperation GP and OHCP	21.6	37	23.9 ^3^	36	27.5	30	23.8	29
Privacy at desk	25.2	38	27.3	40	29.1	40	29.3	30
GP must help me find my way in health care**	17.3 ^3 4^	45	19.0 ^3^^4^	42	25.7 ^1^^2^	38	25.9 ^1 2^	31
Respect of assistant**	44.2 ^2 3 4^	11	32.9 ^1^^3 4^	20	26.8 ^1^^2^	31	20.9 ^1 2^	32
OHCP listens carefully	25.5 ^4^	27	23.2 ^4^	33	22.1	36	15.7 ^1 2^	33
Respect of *HCP* for patient **	42.1 ^3 4^	12	37.1 ^3 4^	14	28.1 ^1^^2^^4^	23	16.3 ^1 2 3^	34
GP must have sufficient time for patient**	29.1 ^4^	24	28.0 ^4^	25	23.3	28	17.6 ^1 2^	35
GP attention for emotional causes of health problems	31.5	25	35.4 ^4^	19	34.6 ^4^	21	25.6 ^2 3^	36
GP must help prevent disease	18.4	49	17.7 ^3^	43	22.2 ^2^	43	17.1	37
GP gives room for participation in decision-making**	28.3 ^2^	28	35.7 ^1^^4^	21	32.3 ^4^	25	22.9 ^2 3^	38
Enough practice information available *	17.0 ^3^	35	21.0	31	24.2 ^1^^4^	33	17.4 ^3^	39
OHCP has information on disease history	19.6	44	18.7	47	22.2	46	17.4	40
GP pays attention to emotional problems*	15.9 ^2 3^	43	22.0 ^1^	35	23.2 ^1^	37	17.6	41
OHCP must spend sufficient time	20.1 ^4^	31	18.1 ^4^	39	16.4	41	10.8 ^1 2^	42
OHCP wants to talk regarding mistakes *	18.9	43	24.0 ^4^	37	24.2 ^4^	39	15.8 ^2 3^	43
OHCP must help prevent disease**	17.4	47	15.1 ^3^	50	23.3 ^2^^4^	47	14.8 ^3^	44
OHCP must motivate advice **	17.7 ^4^	36	15.9 ^3 4^	44	22.2 ^2^^4^	42	8.5 ^1 2 3^	45
GP must redirect when I think it is necessary *	18.4 ^3^	51	18.3 ^3^	48	24.2 ^1^^2^	50	23.3	46
OHCP gives room participation in decision making*	18.1	40	22.3 ^4^	41	21.0 ^4^	44	12.5 ^2 3^	47
Assistant must spend enough time	16.3 ^4^	42	14.0	45	14.6	49	9.7 ^1^	48
Good (fast) contact on telephone **	12.0 ^3 4^	54	13.0 ^3 4^	49	22.5 ^1^^2^	48	22.3 ^1 2^	49
Enough seating in general practice	9.3	53	8.1	51	8.4	52	6.8	50
Quick access to consultation *	11.7 ^3^	48	17.4	43	18.7 ^1^	25	13.1	51
OHCP must pay attention to emotional problems**	12.2 ^3^	52	12.0 ^3^	53	18.3 ^1 2^^4^	50	9.6 ^3^	52
Direct contact with GP	12.8	55	12.5	56	15.3	54	15.1	53
Information on health problems available in practice	14.1 ^4^	50	11.3	54	13.1	53	8.00 ^1^	54
Helped within 15 minutes after the agreed time**	17.3 ^2^^3 4^	46	12.3 ^14^	52	9.5 ^1^	55	7.3 ^1 2^	55
Favourable practice hours	5.7	56	7.2	55	6.6	56	8.0	56
GP prescribes medicine when I think it is necessary	8.8	58	6.9	58	9.4	57	9.2	57
Possibility to go to *AHGPC*	7.1	57	6.9	57	7.8	58	8.6	58

*OHCP* = the Other Health Care Provider within the general practice.

*AHGPC* = After Hours General Practitioner Clinic.

* = *p* < 0.05; ** = *p* < 0.01.

The chi-square tests showed a significant difference in preference score between two or more different age groups for 53.4% (n = 31) of the preference statements. In most cases, patients ‘75 years and older’ had the lowest preference score. The results of the chi-square tests, which compare two groups separately, showed for the group of ‘0-30 years old’, the most significant differences in preference scores (n = 26) with the group of ‘75 years and older’. For the group of ‘30-50 years old’, the most significant differences (n = 28) were also with the group ‘75 years and older’ and the same yields for the group of ‘50-75 years old’ (n = 23).

Table 3 shows the mean number of preference statements which are categorised as ‘very important’ for the different age groups. The results showed that the group of ‘50-75 years old’ had the largest number of preference statements which were categorised as ‘very important’ (mean = 18.1) and the group of ‘75 years and older’ had the lowest mean number of preference statements that were categorised as ‘very important’ (mean = 14.2). This difference was significant (*t*(688) = 3.13; *p* < 0.001).

**Table 3:** Mean number of statements which are ‘very important’ for the different age groups

**Age groups**	**Mean number of statements which are ‘very important’**	**SD**	**N**
0-30 years	17.2	12.1	233
30-51 years	17.7	13.9	514
51-75 years	18.1	14.0	542
75 years and older	14.2	11.2	148

### Logistic regression; controlling for gender, education, perceived health, GP contacts and urbanisation of the practice location

Logistic regressions were conducted for the 31 statements for which the chi-square tests showed a significant difference in the preference scores between the age groups. For most of the statements, the significant relationship between age and preference score did not disappear after the confounders were entered in the logistic regression model. The significant influence of age on preference score disappeared for only five statements after entering the factor education; most of these preference statements concerned the other health care provider. Table 4 shows the five preference statements for which the significant relationship disappeared. The other confounder variables; perceived health, number of GP contacts and GP practice location did not influence the significant relationship between age and preference score.

**Table 4:** Results nested logistic regression analyses for 5 preference statements for which confounders influence the relationship between age and preference score

	**Odds ratio**	**Std. Err.**	**z**	**95% conf.**	**Interval**
Preference statement 36: GP must redirect me to a medical specialist when I think it is necessary					
Reference category 51–75 years					
Model 1					
75 years and older	0.97	0.2	0.87	0.65	1.44
30-51 years	0.72	0.1	0.02	0.55	0.95
0-30 years	0.7	0.13	0.05	0.49	1
Model 2					
75 years and older	0.9	0.18	0.61	0.6	1.35
30-51 years	0.77	0.11	0.06	0.58	1.02
0-30 years	0.72	0.13	0.08	0.5	1.04
Gender	0.93	0.12	0.58	0.73	1.19
Education	0.81	0.07	0.02	0.68	0.96
Preference statement 57: The OHCP* must assist me to prevent diseases or to improve my health					
Reference category 0–30 years					
Model 1					
75 years and older	0.9	0.26	0.7	0.51	1.57
51-75 years	1.57	0.31	0.03	1.06	2.33
30-51 years	0.94	0.2	0.77	0.62	1.42
Model 2					
75 years and older	0.76	0.22	0.35	0.43	1.35
51-75 years	1.46	0.3	0.07	0.98	2.17
30-51 years	0.97	0.21	0.9	0.64	1.48
Gender	1.14	0.16	0.35	0.87	1.49
Education	0.73	0.07	0	0.6	0.89
Preference statement 59 The OHCP must pay attention to emotional problems					
Reference category: 0–30 years					
Model 1					
75 years and older	0.87	0.29	0.68	0.45	1.67
51-75 years	1.71	0.39	0.02	1.09	2.68
30-51 years	1.1	0.26	0.69	0.7	1.76
Model 2					
75 years and older	0.69	0.23	0.28	0.36	1.34
51-75 years	1.57	0.36	0.05	1	2.47
30-51 years	1.16	0.28	0.54	0.72	1.86
Gender	0.99	0.15	0.97	0.74	1.34
Education	0.64	0.07	0	0.51	0.79
Preference statement 62: The OHCP must collaborate well with GP					
Reference category 51–75 years					
Model 1					
75 years and older	0.7	0.13	0.047	0.49	0.99
30-51 years	0.97	0.12	0.82	0.77	1.23
0-30 years	0.96	0.15	0.78	0.71	1.3
Model 2					
75 years and older	0.74	0.13	0.1	0.52	1.06
30-51 years	0.9	0.11	0.39	0.7	1.15
0-30 years	0.91	0.14	0.53	0.67	1.23
Gender	0.91	0.09	0.4	0.74	1.13
Education	1.23	0.1	0.01	1.07	1.43
Preference statement 63: The OHCP must not give conflicting information					
Reference category 0–30 years					
Model 1					
75 years and older	0.66	0.12	0.02	0.46	0.95
51-75 years	0.91	0.11	0.43	0.71	1.16
30-51 years	0.95	0.15	0.73	0.7	1.29
Model 2					
75 years and older	0.75	0.14	0.12	0.52	1.08
51-75 years	0.98	0.12	0.86	0.76	1.25
30-51 years	0.97	0.15	0.84	0.71	1.32
Gender	0.89	0.1	0.27	0.72	1.1
Education	1.2	0.09	0.01	1.04	1.4

*OHCP = The other health care provider.

## Discussion

Health care for the elderly has become an essential part of GP care. In the future, the number of elderly patients with complex health care needs as a result of multi-morbidity, disability, vulnerability, and loss of control will grow [13,23,24]. Despite the complex health care needs of elderly patients, the present study showed no significant difference in the rank order of the 58 preference statements regarding GP between elderly patients and younger patients. Elderly patients find ‘good expertise of GP’, ‘no conflicting information’ and ‘good cooperation’ important quality aspects, just as the other age groups. In this perspective, GPs must pay attention to the same quality aspects for the elderly patients as for the youngest patients.

However, the present study showed differences in the number of preference statements which are categorized as ‘very important’ between the age groups. The elderly patients categorised the lowest number of preference statements as ‘very important’. The fact that the oldest patients are milder has been confirmed by previous research [25] and may be attributed to an age effect. As the life-cycle theory states, age can influence people’s beliefs, values and attitudes regarding health care. The oldest patients become dependent, disabled and develop a loss of self-confidence, which may result, for instance, in less motivation to participate in shared decision making or active information seeking [26]. As a consequence, it may not be necessary to prepare health care providers for an upcoming critical patient group as younger patients grow older.

The present study demonstrated that patient characteristics and practice location did in most cases not confound the significant relationship between age and preference score. Only for five preference statements the factor ‘education’ confounded the relationship between age and preference score. This finding may indicate that older patients with a higher level of education, in some cases, have other preferences regarding quality of GP care than older patients with a lower education level. This may mean that as highly-educated patients grow older, GPs have to be aware of their divergent needs and desires. In this sense, the difference in preference score for the different age groups can be a cohort effect. In the future, GPs may encounter more well-educated elderly people with preferences comparable to those of younger patients [12]. However, education only influenced the relationship for 5 of the 31 preference statements entailing redirection to medical specialists, assistants to prevent diseases, cooperation between health-care providers, conflicting information and attention for emotional problems.

Not only the mean age of the GP’s patient population will change, but also the health care offered by the GP. As patients grow older, GP care will shift from cure to care. Also, the GP care in the Netherlands for patients with a chronic disease will shift to a more patient-centred focus and different disciplines and health-care organisations will be stimulated to cooperate. These changes may change patients’ opinions regarding GP care in the future [27]. Therefore, research into the preferences regarding GP care for different patient groups should be repeated in the future.

A limitation of this study, given the number of comparisons made and statistical tests performed, is the issue of multiple testing. In short, the multiple testing issue entails that when a series of comparisons are performed while in reality there are no differences, 5% of these test will show a significant difference solely due to chance. Statistical solutions to this problem, such as the Bonferroni correction for example, generally reduce power. Accordingly, we chose not to apply a statistical correction for multiple comparisons, but to address this issue when interpreting the results. In this context, it is worth noting that although we found much more significant results than could be expected based on chance alone, a small number of the significant results may potentially be a result of the number of tests performed.

A second limitation is the arbitrary approach by which the age groups have been categorised, especially, the age group 0–30 years old which include preferences from parents with young children with preferences from young adults. Nevertheless, our data show that the group of ‘0-30 years old’ had the most significant differences in preference scores (n = 26) with the group of ‘75 years and older’. For the group of ‘30-50 years old’, the most significant differences (n = 28) are also with the group ‘75 years and older’ and the same is true for the group ‘50-75 years old’ (n = 23). So, the most divergent group is the age group ‘75 years and older’. Previous research using other age groups confirmed that preference scores differ between age groups. In addition, we found that generally the relationship between age and preference score is not significantly influenced by patient and practice characteristics.

Another aspect which has to be taken into account is the fact that patient preferences are influenced by the length of time that elapsed between the consultation and filling in the survey [28]. We have no information regarding the length of time elapsed between the consultation and the survey. However, our sample contained people who had visited their GP in the year preceding the survey and people who had not visited their GP in that year. Therefore, the preference scores were not only influenced by recent experiences of our sample.

Lastly, our study did not investigate every possible patient or practice characteristic. For example, patients’ religion may also influence the relationship between age and preference score. According to a systematic review, religion is most frequently found to influence patients’ preferences [6]. However, our data set did not include this variable.

One of the strengths of our research is the large number of preference statements which were investigated, that these preference statements were based on interviews and focus groups with patients and were approved by different health-care organisations. Moreover, our survey was developed using widely known and tested CQI-methodology [15] and also included preference statements regarding the other health care provider in GP care which is rather unique.

## Conclusion

The present study investigated the preferences regarding general practice care of elderly patients and whether patient characteristics and practice location may confound the relationship between age and the categorisation of a preference score as very important. This study demonstrated that the preferences of elderly patients concerning GP care concern the same items as younger patients. However, their preferences are less strong, which cannot be ascribed to gender, education, perceived health, the number of GP contacts and practice location.

### Ethical approval

Not required since published data were used.

## Competing interests

The authors declare that they have no competing interests.

## Authors’ contributions

WAdGR, AJB, DdB and DHdB contributed to the design of this study. AJB was responsible for the subject of this study. WAdGR was responsible for the day-to-day management, the statistics and produced the first draft of the manuscript. All authors contributed to the write-up of this study. All authors read and approved the final manuscript.

## Pre-publication history

The pre-publication history for this paper can be accessed here:

http://www.biomedcentral.com/1471-2296/14/90/prepub
